# A fast and inexpensive genotyping system for the simultaneous analysis of human and *Aedes albopictus* short tandem repeats

**DOI:** 10.1186/s13071-023-05977-w

**Published:** 2023-10-05

**Authors:** Andreu Albó Timor, Federica Lucati, Frederic Bartumeus, Jenny Caner, Santi Escartin, Simone Mariani, John R. B. Palmer, Marc Ventura

**Affiliations:** 1 Centre for Advanced Studies of Blanes (CEAB-CSIC), Blanes, Spain; 2 Department of Political and Social Sciences, Universitat Pompeu Fabra (UPF), Barcelona, Spain; 3 Centre for Research on Ecology and Forestry Applications (CREAF), Cerdanyola del Vallès, Spain; 4 Catalan Institution for Research and Advanced Studies (ICREA), Barcelona, Spain

**Keywords:** *Aedes albopictus*, Blood meal analysis, Human fingerprinting, STR genotyping, Vector-host interaction

## Abstract

**Background:**

Determination of the interactions between hematophagous mosquitoes and their human hosts is of great importance for better understanding the transmission dynamics of mosquito-borne arboviruses and developing effective strategies to mitigate risk. Genetic analysis of human and mosquito DNA can play a key role in this, but commercial kits for human short tandem repeat (STR) genotyping are expensive and do not allow for the simultaneous STR analysis of host and vector DNA. Here, we present an inexpensive and straightforward STR-loci multiplex system capable of simultaneously amplifying *Aedes albopictus* and human STRs from blood-fed mosquitoes. Additionally, we examine the effect of storage methods and post-feeding time on the integrity of host DNA.

**Methods:**

Thirty-five STRs (16 human and 19 *Ae. albopictus* STRs) subdivided in three multiplexes were tested for amplification and scoring reliability. Under laboratory conditions we compared the efficacy of two preservation methods (absolute ethanol vs lysis buffer) on the integrity of host DNA in *Ae. albopictus *blood meals. We also evaluated the effect of post-feeding time by sacrificing blood-fed mosquitoes at different time intervals after feeding, and we assessed our ability to detect multiple feedings. To determine if the system can be employed successfully under field conditions, we carried out a preliminary study using field-collected *Ae. albopictus*.

**Results:**

All 35 STRs amplified consistently in the laboratory. Lysis buffer performed better than absolute ethanol in terms of allele peak height and clarity of electropherograms. Complete human DNA profiles could be obtained up to 48 h following the blood meal. Analysis of multiple feedings confirmed that peak heights can be used as a proxy to determine post-feeding time and thus derive the number of different people bitten by a mosquito. In the field trial, amplification was successful for 32 STRs. We found human DNA signal in 38 of the 61 field-collected mosquitoes (62%), of which 34 (89%) had ingested a single blood meal, while four (11%) contained double meals.

**Conclusions:**

Our new genotyping system allows fast and reliable screening of both host and vector species, and can be further adapted to other mosquito species living in close contact with humans.

**Graphical Abstract:**

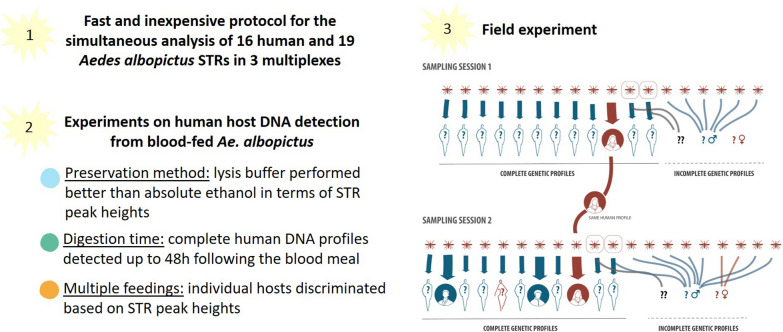

**Supplementary Information:**

The online version contains supplementary material available at 10.1186/s13071-023-05977-w.

## Background

The Asian tiger mosquito, *Aedes albopictus*, is an invasive disease vector that constitutes a major public health concern, mainly because of its ability to transmit a variety of arboviruses, including dengue, Zika and chikungunya [1–3]. *Aedes albopictus* is considered to be one of the world’s most invasive species because, although originally native to the tropical forests of Southeast Asia, in the late 1970s it started to spread rapidly to a wide range of temperate and tropical regions across the globe [4]. Taken together, the rapid geographical spread of *Ae. albopictus* and its potential to vector a wide range of pathogens present a challenge for global economies and a threat to public health [5–7]. This, together with its tendency to live in highly populated and urban areas, its anthropophagic [8–10] and aggressive daytime human-biting behavior [11] and its tendency to ingest multiple blood meals during each gonotrophic cycle [12], amplify the species’ potential to transmit pathogens through the human-mosquito networks formed through each bite.

Studying the characteristics of these networks of mosquito-human interactions is of great importance for better understanding the dynamics of mosquito-borne diseases and developing effective strategies to mitigate risk. Traditionally, mosquito identification was based on morphological characters. However, the need to better understand interactions between arthropod vectors and their vertebrate hosts, coupled with the advent of molecular biology methods during the twentieth century, revolutionized this field. These developments enhanced capabilities for species identification and made it possible to detect vertebrate host DNA in mosquito blood meals [13, 14]. Indeed, during the early 1990s, PCR-based amplification of variable fragments of host DNA from mosquito blood meals significantly improved the identification of vertebrate hosts [15, 16], and it soon became one of the best ways to study the feeding patterns of mosquitoes that tend to feed on human blood [17]. More concretely, short tandem repeats (STRs) have become more and more widely used in human DNA blood meal profiling due to their capacity to recover information from low amounts of DNA, their high degree of discrimination, their cost effectiveness and their compatibility with multiplexing methods [18]. Consequently, in the last decades, studies targeting human STRs for DNA fingerprinting from blood-fed mosquitoes have flourished, making it possible to examine mosquito feeding patterns in a very precise way [19–22]. STRs are also widely employed in the characterization of genetic diversity and structure of mosquito vector species, as well as in the delineation of their invasion routes [23–25]. Simultaneous and inexpensive genetic analysis of human and mosquito DNA could provide a fuller and more detailed picture of the transmission dynamics of mosquito-borne arboviruses and, thus, could be valuable in the design of targeted policy interventions for reducing disease risk. Nevertheless, commercial kits commonly used for human STR genotyping are expensive and do not allow for the simultaneous analysis of host and vector STR loci (e.g. [26]).

In this study, we present for the first time an inexpensive and straightforward STR-loci multiplex system capable of simultaneously amplifying 19 *Ae. albopictus* and 16 human STRs, including the sex determinant locus amelogenin, from blood-fed mosquitoes. Additionally, we examine four issues that present possible limitations to the system: (i) whether our set of human STR loci allows for discrimination of closely related individuals; (ii) the differential efficacy of two of the most commonly used preservation methods (absolute ethanol and lysis buffer) on the integrity of human DNA extracted from blood-fed *Ae. albopictus*; (iii) the time interval over which human DNA can be detected in a blood meal before the information is lost due to DNA degradation and digestion; and (iv) whether we can determine the number of different people in a single blood meal and whether human DNA from the blood meal can be matched to that found in the same person’s saliva. Finally, to establish whether the system can be employed successfully under field conditions, we present a preliminary field study in which we used blood-fed mosquitoes collected in Catalonia (northeastern Iberian Peninsula). Although this new genotyping system was designed for use on *Ae. albopictus*, it can be further adapted to other mosquito species living in close contact with humans.

## Methods

### Sampling, DNA extraction, microsatellite screening and analysis

#### Blood-fed mosquitoes

*Aedes albopictus* individuals were collected as eggs or larvae during 2020 and reared in captivity. To this purpose, standard ovitraps were placed in the Marimurtra Botanical Garden in Blanes (Spain; see Additional file 1: Figure S1). The ovitraps consisted of dark plastic containers filled with water, with a thin wooden blade as oviposition support (the egg-collector), following the methodology used in the pan-European *Aedes* Invasive Mosquito species (AIM) COST Action and other surveillance programs [27]. In the laboratory, egg-collectors with variable numbers of tiger mosquito eggs were placed in tap water at around 21 ºC for larvae to hatch. Alternatively, larvae were captured directly from storm drains located at roads in the surroundings of the Marimurtra Botanical Garden. All samples were kept in the Live Organism Experimental Laboratory (LEOV) of the Center for Advanced Studies of Blanes (CEAB-CSIC, Blanes; see Additional file 1: Figure S1). Adult *Ae. albopictus* were placed in a screened bug dorm cage under natural day-night light conditions at constant temperature (about 21 °C). No blood was provided prior to the experiments, and mosquitoes were fed on either cotton discs imbued with a solution of tap water and 10% sugar or apple slices of approximately 20 g, which were replaced every 2 days. On days 3 to 5 from hatching, females were allowed to feed to repletion on a human arm (of one of the authors). After feeding, engorged females were collected directly from the host arm using an electric insect aspirator. All samples (a total of 50 engorged mosquitoes) were saved for DNA analyses by placing them into 1.5 ml microcentrifuge tubes with a preservation agent (absolute ethanol or lysis buffer) and stored at − 80 °C.

Mosquito and host DNA was extracted using a slightly modified version of the DNeasy Blood and Tissue kit (Qiagen, Hilden, Germany) protocol. Abdomens of engorged females were isolated from the rest of the body to avoid degradation of DNA by the endonucleases contained in the eyes [13], and macerated using forceps and tips, releasing the blood into the lysis solution. Forceps were sterilized and air dried between each mosquito to prevent cross contamination of samples. The samples were then incubated at 56 °C for 24 h with proteinase K before proceeding with the protocol, which finished with a two-stage DNA elution of 15 µl each with DNA-free milliQ water (final elution volume of 30 µl).

We simultaneously amplified 16 human STRs (including 13 CODIS loci, two STRs commonly used for human fingerprinting and the gender identification locus amelogenin [26]) and 19 STRs specific for *Ae. albopictus* [28, 29]. The analyzed STRs are described in Table 1. Loci were selected based on their easy and clear characterization and high variability. We developed a novel multiplex organization to amplify simultaneously human and mosquito microsatellites, which consisted of a cost-effective and efficient protocol based on multi-colored fluorescent primers (fluorophores 6-FAM, Hex, Atto560 and Atto565 for mosquitoes; 6-FAM, Joe, Tamra and Atto565 for humans) spread across three multiplexes (Table 1). The program Multiplex Manager 1.2 [30] was used to plan and optimize this distribution. Multiplex PCR amplifications were performed in a reaction volume of 11 µl, containing 5.5 µl Qiagen multiplex PCR master mix, 1.0 µl genomic DNA, 1.0 µl Q-solution and variable volumes of primer mix and water. The PCR thermocycling program consisted of 95 °C for 11 min; 30 cycles at 94 °C for 30 s, 60 °C for 1 min and 72 °C for 1 min; and a final extension at 60 °C for 30 s. PCR products were run in 1.2% agarose gels and visualized under UV light at 300 nm.

**Table 1:** Combined human-*Aedes albopictus* short tandem repeats set employed in the present study

Locus	Repeat motif	Allele size range (bp)	Primer sequences	C_0_ (μM)	MPX	Primer label (fluorescent dye) for blood-fed mosquitoes	Primer label (fluorescent dye) for buccal swabs
Alb-tri-3^a^	[AGA]n	123–153	F: AGATGTGTCGCAATGCTTCC R: GATTCGGTGATGTTGAGGCC	0.3	1	6-FAM	
Aealbmic11^b^	[TGT]n	188–230	F: CTCTGCGTTCCGGTTCTATC R: AGGCAACCTCTCGAATGAAA	0.3	1	6-FAM	
Aealbmic4^b^	[CAA]n	167–183	F: ATCGCGGGTTTTCTATTCCT R: ATCAACGAAACCGAAAGCAT	0.3	1	6-FAM	
Aealbmic13^b^	[GAT]GAC [GAT]n	132–171	F: TCACACCATGGTCAAAGCAT R: TGCTGAGTTGAATGGAAACG	0.3	1	HEX	
Alb-tri-18^a^	[ACA]n	250–280	F: ACACAATTGCCGTTCAGCTC R: CGTCTAATAGCTCCGGTCCC	0.3	1	HEX	
PentaD^c^	[AAAGA]n	376–449	F: GAAGGTCGAAGCTGAAGTG R: ATTAGAATTCTTTAATCTGGACACAAG	0.3	1	JOE	JOE
D3S1358^c^	[AGAT]n, [TCTA]n	115–147	F: ACTGCAGTCCAATCTGGGT R: ATGAAATCAACAGAGGCTTGC	0.25	1	TAMRA	6-FAM
Aealbmic12^b^	[GAT]n	155–182	F: AGAGCCCTCGAAAAGAGAGC R: AGCACTCATTCTTGGCTTGG	0.3	1	ATTO550	
Aealbmic3^b^	[AAC]n	200–239	F: ACCATACAGCCTGGAGTTCG R: GGGGTTGTGTGAATTGTCGT	0.3	1	ATTO550	
FGA^c^	[TTTC]n	322–444	F: GGCTGCAGGGCATAACATTA R: ATTCTATGACTTTGCGCTTCAGGA	0.3	1	ATTO565	TAMRA
Alb-tri-21^a^	[AGGG]n	137–206	F: AGGGCTTCAATGGGTCTCTC R: TGGTTATTAATACGGCGAGGC	0.5	1	ATTO565	
Aealbmic7^b^	[TTG]nATG[TTG]n	194–215	F: ATAGACGGGAGTCGGTTCCT R: TCCAACCGCTAGTGTCATCA	0.3	1	ATTO565	
Aealbmic5^b^	[TGT]n	136–214	F: AACCCATCGAACACAGAAGG R: GTACGGTTGACTCGCTGTGA	0.3	2	6-FAM	
D18S51^c^	[GAAA]n	290–366	F: TTCTTGAGCCCAGAAGGTTA R: ATTCTACCAGCAACAACACAAATAAAC	0.25	2	6-FAM	6-FAM
PentaE^c^	[AAAGA]n	379–474	F: ATTACCAACATGAAAGGGTACCAATA R: TGGGTTATTAATTGAGAAAACTCCTTACAATTT	0.25	2	6-FAM	6-FAM
Alb-tri-46^a^	[TTC]n	158–192	F: TTCACAACATACGGAATCGC R: GGTCCGGTGTAATAGCCTCC	0.3	2	HEX	
D16S539^c^	[GATA]n	264–304	F: GGGGGTCTAAGAGCTTGTAAAAAG R: GTTTGTGTGTGCATCTGTAAGCATGTATC	0.2	2	JOE	JOE
Aealbmic9^b^	[GAT]n	128–143	F: GCGATGACAGTGGAACAAGA R: GCTTGGCAGGGAACAAATTA	0.5	2	ATTO550	
Alb-tri-20^a^	[GTG]n	165–201	F: GTGCCGTTGATCATCCTGTC R: TCCAGCACCGTGAGTAATCC	0.3	2	ATTO550	
Alb-tri-25^a^	[CCAA]n	257–278	F: CCAACCAACAACCCAGGAAC R: TACGATGCGCAACCATCATC	0.3	2	ATTO550	
D5S818^c^	[AGAT]n	119–155	F: GGTGATTTTCCTCTTTGGTATCC R: AGCCACAGTTTACAACATTTGTATCT	0.1	2	ATTO565	ATTO565
Alb-tri-44^a^	[CAC]n	173–212	F: CACTCGCGCGTGTTCTTC R: GACGCACCATCAGCATCATC	0.3	2	ATTO565	
CSF1PO^c^	[AGAT]n	321–357	F: CCGGAGGTAAAGGTGTCTTAAAGT R: ATTTCCTGTGTCAGACCCTGTT	0.3	2	ATTO565	ATTO565
Alb-tri-45^a^	[TTT]n	120–150	F: TTTCAGCTCGGTGTTATGGC R: TGATGTTGATGATGATGACTACGA	0.3	3	6-FAM	
Alb-tri-6^a^	[AGC]n	164–219	F: AGCACGAGTACAGAATGTGC R: TGGCCTCCTACCGTTTATCTG	0.3	3	6-FAM	
D21S11^c^	[TCTA]n, [TCTG]n	203–259	F: ATATGTGAGTCAATTCCCCAAG R: TGTATTAGTCAATGTTCTCCAGAGAC	0.25	3	6-FAM	6-FAM
Alb-tri-33^a^	[GGC]n	137–182	F: GGCTGCTGTTGTTGGTACG R: CACGTTCAATCACCGGTTCC	0.3	3	HEX	
D13S317^c^	[GATA]n	176–208	F: ATTACAGAAGTCTGGGATGTGGAGGA R: GGCAGCCCAAAAAGACAGA	0.25	3	JOE	JOE
D7S820^c^	[GATA]n	215–247	F: ATGTTGGTCAGGCTGACTATG R: GATTCCACATTTATCCTCATTGAC	0.25	3	JOE	JOE
Amelogenin^c^	–	106–112	F: CCCTGGGCTCTGTAAAGAA R: ATCAGAGCTTAAACTGGGAAGCTG	0.3	3	TAMRA	TAMRA
vWA^c^	[AGAT]n	123–171	F: GCCCTAGTGGATGATAAGAATAATCAGTATGTG R: GGACAGATGATAAATACATAGGATGGATGG	0.25	3	TAMRA	TAMRA
D8S1179^c^	[TATC]n	203–247	F: ATTGCAACTTATATGTATTTTTGTATTTCATG R: ACCAAATTGTGTTCATGAGTATAGTTTC	0.25	3	TAMRA	TAMRA
Alb-tri-41^a^	[GAT]n	134–155	F: GATCGATTTGGGAGCTTCTG R: GAACCTCTTCTCGCTTGGCT	0.3	3	ATTO565	
TH01^c^	[AATG]n	156–195	F: GTGATTCCCATTGGCCTGTTC R: ATTCCTGTGGGCTGAAAAGCTC	0.2	3	ATTO565	ATTO565
TPOX^c^	[AATG]n	262–290	F: GCACAGAACAGGCACTTAGG R: CGCTCAAACGTGAGGTTG	0.2	3	ATTO565	ATTO565

The repeat motif, allele size range, forward (F) and reverse (R) primer sequences, primer concentration (C_0_), amplification multiplex panel (MPX) and primer label are shown for each short tandem repeat (STR)

^a^*Aedes albopictus* STRs from Beebe et al.[29]

^b^*Ae. albopictus* STRs from Manni et al.[28]

^c^Human STRs [26]

#### Saliva

Human DNA extracted from the saliva of 12 study participants was used to obtain reference allelic profiles, which were stored in an anonymized database. After each participant provided informed consent, samples were collected by swabbing the inner cheek with a sterile applicator stick for approximately 30 s. The swabs were taken and mixed gently into a 15 ml Falcon tube with 500 µl of phosphate buffered saline (PBS) solution and preserved at − 20 °C. Human samples were given unique numerical codes to decouple them from subjects’ identities.

DNA extraction from saliva followed a similar protocol as for the blood-fed mosquitoes, except for the incubation time, which lasted only 10 min for the saliva. Moreover, after removing the applicator stick from the Falcon tube, the solution containing DNA was cleaned twice to obtain a higher purification, first with 500 µl of PBS solution and then with 250 µl of PBS solution, as proposed by Ng et al. [31]. From this step onward, the protocol described above was followed, with final elution volumes of 100 µl for each sample.

The same 16 human STRs were distributed in one multiplex and amplified under the previous conditions (Table 1). To test the discriminatory power provided by our microsatellite set, we took oral swabs from volunteers in two families of four and five members (each composed of a mother, a father and two or three adult siblings), who provided informed consent. Principal coordinates analysis (PCoA) of the obtained genetic profiles was used to determine whether each family member had a unique DNA fingerprint and to visualize genetic differences between profiles [32]. The PCoA was run using the ‘pcoa’ function in the ape package 5.3 [33] in R 4.1.0 [34], starting from a Nei’s D_A_ genetic distance matrix, which was created through the MSA program [35]. Sample size was chosen based on sample availability and published literature [36].

#### Microsatellite genotyping and sizing

Genotyping of the PCR products was performed by Secugen S.L. (Madrid, Spain) on a 48-capillary 3730xl DNA Analyzer (Applied Biosystems, Thermo Fisher Scientific, Waltham, MA, USA). Fragments were sized with GeneScan LIZ-500 size standard (Thermo Fisher Scientific) and binned using Geneious 11.1.5 [37].

#### Computer-assisted identification of profiles

To check if human allelic profiles extracted from engorged mosquitoes came from any of the participants who provided saliva, one Nei’s D_A_ genetic distance matrix (including both human profiles derived from engorged mosquitoes and those derived from cheek swabs) was created from allelic data through the MSA program, and samples were compared using MS Excel (Microsoft Corp., Redmond, WA, USA). Only pairs of samples with D_A_ distance < 0.15 were considered as possible matches; this was done to account for possible amplification failures.

### Accuracy and limits for human host DNA detection in blood-fed mosquitoes

Three experiments were conducted on laboratory-reared engorged mosquitoes, with the aim to: (i) compare the efficacy of two preservation methods, namely absolute ethanol versus lysis buffer, on the integrity of human DNA; (ii) determine the time interval over which human DNA can be detected in a blood meal; and (iii) understand our ability to discriminate between multiple feedings from a single mosquito. For this last point, human DNA profiles extracted from blood meals were compared with reference profiles obtained from buccal swabs. Sample sizes were chosen based on sample availability and published literature [e.g. 20, 30], with the goal of maximizing statistical power subject to logistical constraints. Statistical analysis was performed using one-way analysis of variance (ANOVA) followed by Tukey’s test.

#### Host DNA detection capacity according to the preservation method

Five engorged mosquitoes were preserved using each preservation method: absolute ethanol and lysis buffer (40 mM EDTA; 50 mM Tris pH 8.3; 0.75 M sucrose). The time between mosquito collection and preservation was < 5 min. Success rate of amplification and allele detectability ─ determined by comparing electropherogram peak heights (PHs) ─ were assessed to compare the two preservation methods. Regarding PHs, homozygotes were assumed to present a single peak of twice the heterozygote PH for a single allele. In addition, mean PHs throughout all loci were calculated as the sum of the PHs for the alleles detected in each sample divided by the total number of alleles detected, and standardized following Sneath and Sokal [38].

#### Digestion time limits for detecting host blood

A total of 21 engorged *Ae. albopictus* were sacrificed at different times after blood feeding: four mosquitoes at 0 h, 12 h, 24 h and 48 h; three at 72 h; and two at 96 h. All were stored in absolute ethanol at − 80 °C until DNA extraction. To determine the evolution of DNA detectability over time, we analyzed the rate of detectability of each locus and the mean PHs at the different times post-feeding.

#### Capacity for detecting human host DNA in multiple feeding cases

Seven *Ae. albopictus* adults were offered partial (i.e. incomplete) blood meals from human hosts (all authors of this study): two of these mosquitoes were offered meals from two hosts and five were offered meals from three hosts. For each mosquito, time between consecutive meals was < 5 min. Samples were stored in lysis buffer at - 80 ºC. The number of different people in a single blood meal was determined by examining the number of alleles detected at the different human STR loci.

### Field validation of the protocol

A preliminary field study was carried out to test the reliability and feasibility of our combined human-mosquito microsatellite set.

#### Field site and sample collection

The study was conducted in the Marimurtra Botanical Garden (Blanes, Girona; 41°40′36.6″N, 2°48′08.5″E; 4 ha) and its surroundings (see Additional file 1: Figure S1). This is a suitable place for the establishment and proliferation of *Ae. albopictus* due to the high variability of vegetation, temperate climate, abundance of water and high humidity. Sample collection was conducted in two periods of time separated by 3 months: late spring (HMIP-A) and early autumn (HMIP-B) 2021, when mosquitoes abound and the garden is visited by a high number of tourists. In total, 61 *Ae. albopictus* mosquitoes were captured using three different methods: (i) entomological aspirators; (ii) Biogents BG Sentinel 2 traps (Biogents AG, Regensburg, Germany); and (iii) direct catches performed by volunteers and collaborators, who were provided with a small plastic container with a top for mosquito collection. At the same time, the cheek swabbing procedure described in section Saliva was used to collect saliva samples from 40 volunteers and collaborators, all of whom first provided informed consent. As a precaution against SARS-CoV-2 infection, given the ongoing pandemic at that time, each participant swabbed his/her own inner cheek with a sterilized stick. All research collaborators provided oral swabs in order to make it possible to check for sample contamination during mosquito collection or laboratory analysis. Following collection, mosquitoes (preserved in lysis buffer) and cheek swabs (preserved in PBS) were saved for DNA analysis under the conditions described in section Blood-fed mosquitoes and other sections.

#### Genetic analyses

Mosquito and human host DNA was extracted, amplified and profiled as described above. Possible genetic structure of both mosquito and human DNA profiles was evaluated through PCoA following the procedure described in section Saliva.

## Results

### STR amplification and discriminatory power

A total of 32 STR loci (16 human and 19 *Ae. albopictus* STRs) combined in three multiplexes were simultaneously amplified (Table 1). All loci amplified consistently and were polymorphic, with a mean number of alleles per locus of 6.7 in human samples and 4.4 in *Ae. albopictus* samples.

**Fig. 1:**
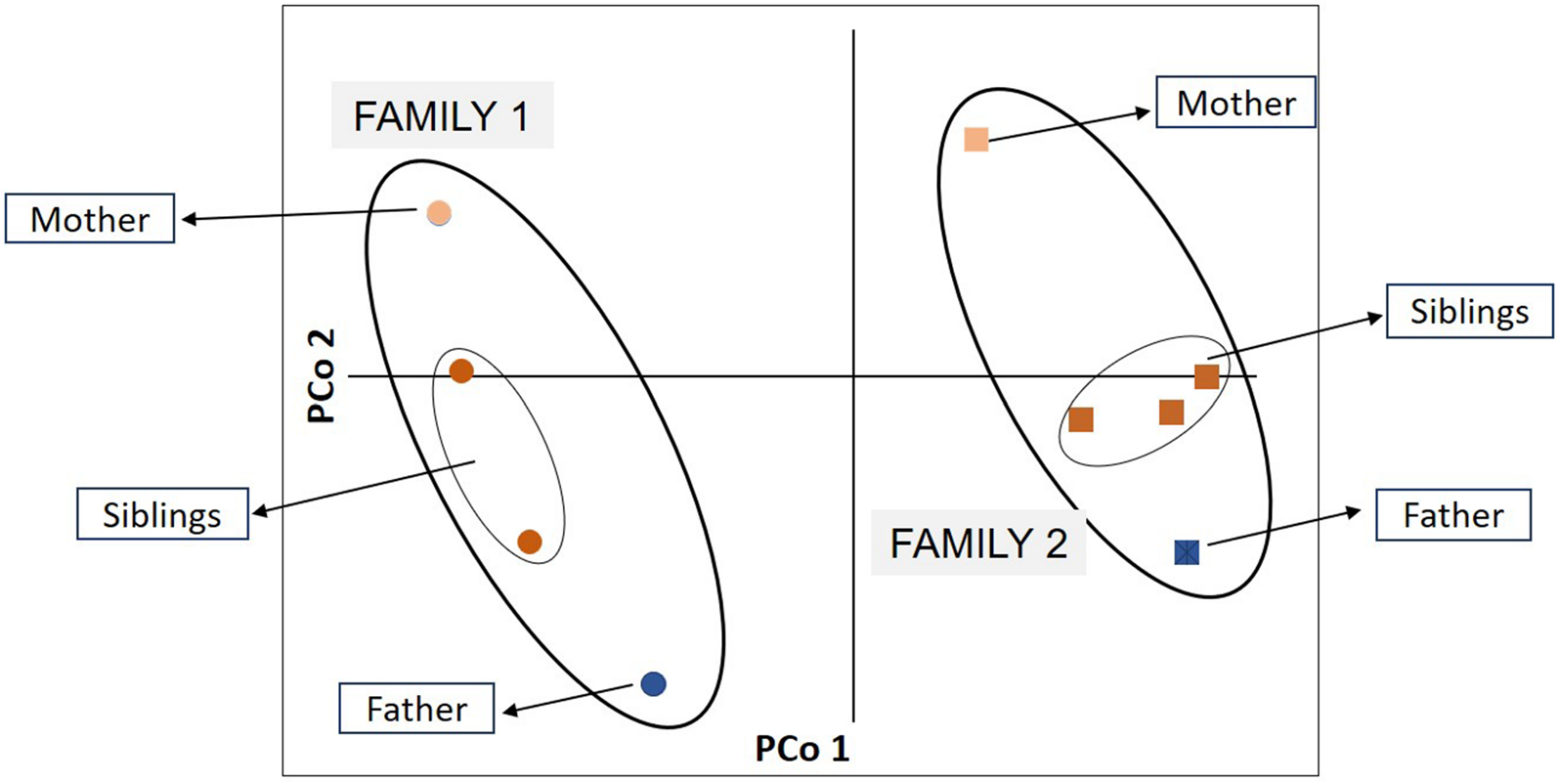
Principal coordinates analysis showing genetic differentiation between members of two family nuclei screened for 16 human short tandem repeats. PCo, Principal coordinate

The two-family nuclei from whom cheek swabs were obtained could be clearly differentiated by PCoA analysis (Fig. 1). Within each family, each member had a unique allelic profile and could be clearly differentiated from the others, with the siblings having DNA profiles intermediate between those of the parents.

### Accuracy and limits for host DNA detection

#### Sample preservation

**Fig. 2:**
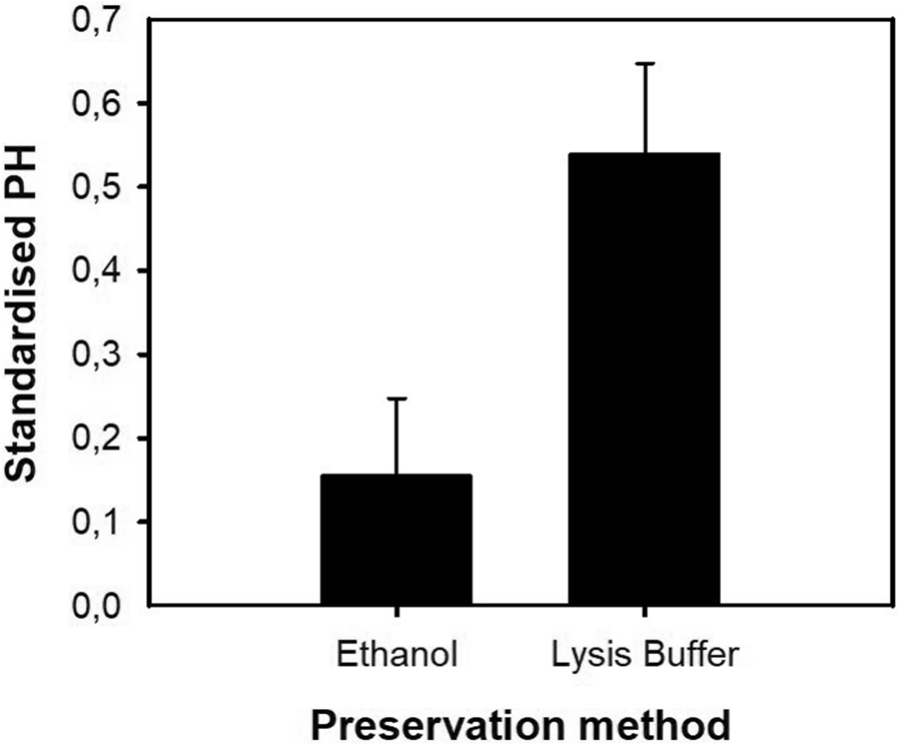
Correlation between preservation method (absolute ethanol vs lysis buffer) and average standardized PHs based on 16 human STRs. Mean values and standard deviations of average PHs are shown. PHs, Peak heights

Of the 10 engorged mosquitoes analyzed, one (preserved in ethanol) was excluded from the analysis due to possible contamination during DNA extraction. Overall, with both preservation methods we detected a 100% allele amplification rate in all loci at 0 h of digestion. However, after applying the standardization proposed by Sneath and Sokal [38], mean PHs were higher in all studied loci in samples preserved with lysis buffer (Fig. 2). On average, PHs obtained from samples preserved with lysis buffer were 120% higher than the ones from samples preserved with ethanol. One-way ANOVA corroborated that the differences in PHs between both methods were significant (ANOVA, *F*_(1, 62)_ = 236, *P* < 0.0001).

#### Digestion time limits for detecting host blood

**Fig. 3:**
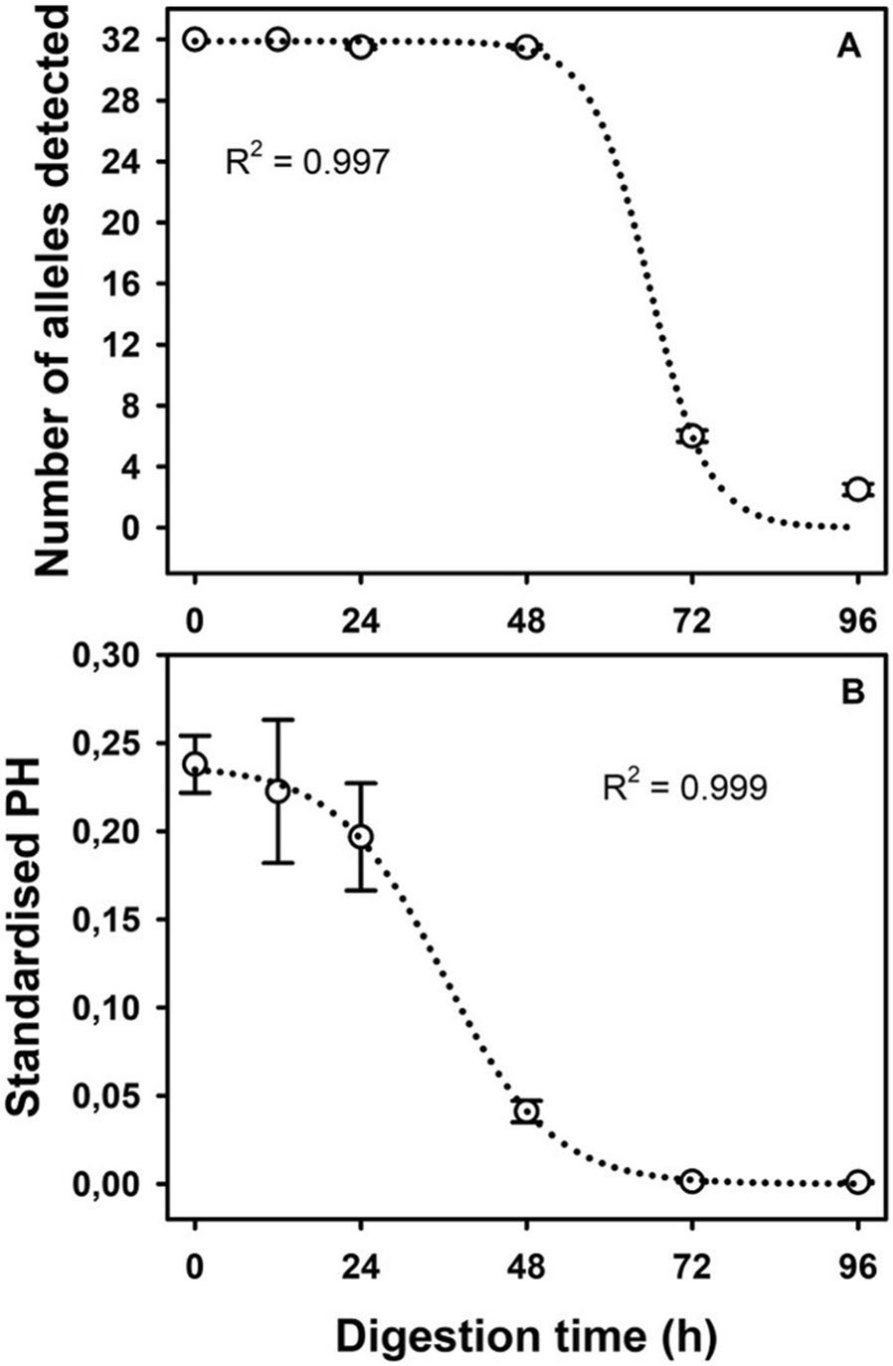
Correlation between post-feeding time (h) and mean number of alleles detected (**a**) and average standardized PHs of the detected alleles (**b**). Empty circles indicate the mean values of average PHs detected at each post-feeding time, together with their standard deviations (whiskers). Dotted lines indicate the fitted sigmoid curves. PHs, Peak heights

On average, nearly 100% of human alleles (31.5 out of 32 possible alleles) were detected in the analyzed blood meals from 0 to 48 h after feeding (Fig. 3a). After 48 h the number of alleles rapidly decreased over time to the point that < 20% of alleles were detectable at 72 h; at 96 h, host DNA was almost undetectable. The evolution of standardized PHs with digestion time follows a descending sigmoid curve (Fig. 3b). Standardized PHs were highest from 0 to 24 h post-feeding; subsequently, standardized PHs sharply decreased and were 0 at 72 h post-feeding. One-way ANOVA showed significant differences between post-feeding times (ANOVA, *F*_(5, 330)_ = 19.42, *P* < 0.0001), and Tukey’s test revealed significant differences in standardized PHs between groups 0–24 h and 48–96 h post-feeding (*P* < 0.0001).

#### Capacity for detecting human host DNA in multiple feeding cases

Seven lab-reared mosquitoes were offered partial blood meals from either two or three different hosts (all authors of this study), with meals separated by < 5 min. In general, full or partial profiles of all hosts were detected in all mosquitoes. In all but one mosquito, individual allelic profiles could be correctly discriminated based on the relative PHs of the 16 human STRs, which is a proxy for DNA template amount [39]. We found only one case where the relative PHs of two hosts in a single blood meal were similar. In every sample, full allelic profiles from the host with highest PHs were correctly detected and assigned to the relevant homologous profiles stored in our saliva database (see Methods). Only partial profiles were detected for the other hosts.

### Field evaluation

#### Human DNA

**Fig. 4:**
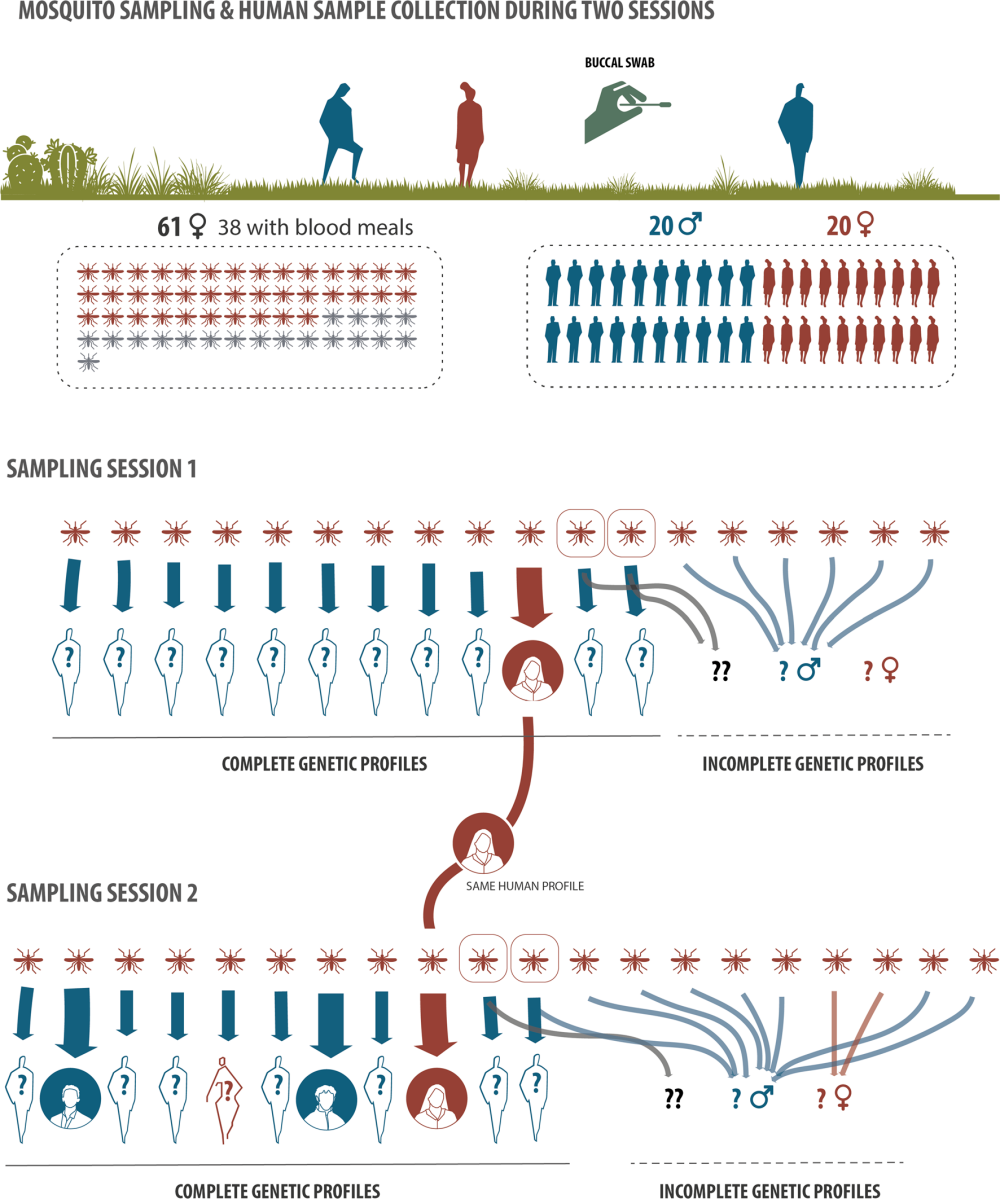
Summary figure of the field trial and human-mosquito biting networks identified in the two sampling sessions. Of the 61 field-collected *Aedes albopictus*, human DNA signal was found in 38 (top left). Buccal swabs were collected from 40 individuals to look for possible matches with human DNA profiles from mosquitoes (top right). As shown in the biting networks (sampling sessions 1 and 2), 34/38 mosquitoes had ingested a single blood meal, while four (circled in red) contained double meals, totaling 42 human genetic profiles. Of the 42 human profiles, 23 were complete or nearly complete (continuous line below networks), while 19 were incomplete (dashed line). Of the 40 profiled individuals, three (depicted inside filled circles in the biting networks) were bitten by four different mosquitoes

**Fig. 5:**
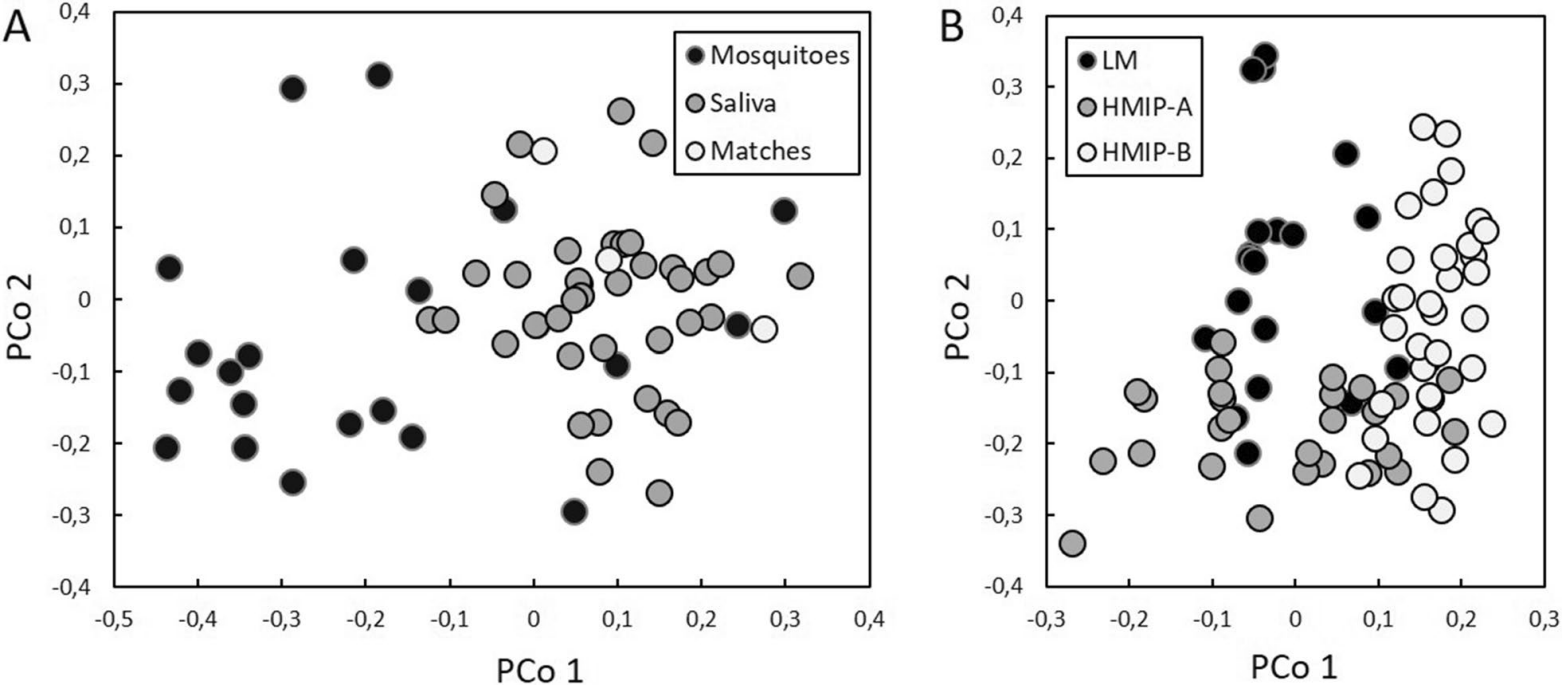
Principal coordinates analysis for human genetic profiles (**a**) and *Aedes albopictus* mosquitoes (**b**). **a** Complete and nearly complete human genetic profiles retrieved from field-collected blood-fed *Ae. albopictus* are shown in black, profiles obtained via oral swabbing are shown in gray and the three cases of human profiles obtained via oral swabbing matching those retrieved from field-collected mosquitoes are shown in white. **b** Colors (black [LM], grey [HMIP-A], white [HMIP-B]) represent the mosquito collection period. HMIP-A, field-collected mosquitoes in late spring 2021; HMIP-B, field-collected mosquitoes in early autumn 2021; LM, laboratory-reared mosquitoes collected as eggs or larvae in 2020; PCo, Principal coordinate

 Complete allelic profiles at all loci were retrieved from all 40 individuals sampled by oral swabbing, and all profiles were unique (i.e. each individual had a unique and specific combination of alleles). Regarding the 61 collected mosquitoes, of the initial set of 16 human STRs, only 13 were considered for analyses, as locus PentaD failed to amplify in most samples and the allele size ranges of loci vWA and D5S818 were found to overlap with those of other STRs. Human DNA signal was found in 38/61 (62%) mosquitoes, of which 34 (89%) had ingested a single blood meal, while four (11%) contained double meals, totaling 42 human profiles (Fig. 4). Nevertheless, only 23 (55%) out of the 42 retrieved human profiles were complete or nearly complete (i.e. amplification was successful for ≥ 11 loci). When considering the 23 nearly complete profiles only (which were found in 23 different mosquitoes; see continuous line below networks in Fig. 4), four matched three profiled humans (depicted inside filled circles in the biting networks of Fig. 4), i.e. three profiled humans were bitten by four different mosquitoes (as one profiled female was bitten by two mosquitoes; Figs. 4, 5a). The human-mosquito biting network derived from the 42 profiles is represented in Fig. 4.

#### Mosquito DNA

 All 61 sampled mosquitoes amplified consistently. We identified an average of 37.44 alleles per sample (standard error [SE] ± 0.18) out of the 38 possible ones. PCoA analysis revealed three partially overlapping groups (Fig. 5b): a first group consisted of mosquitoes sampled as eggs or larvae in 2020 and reared in the laboratory (LM), whereas the second and third groups consisted of mosquitoes collected in 2021 during the field study (HMIP-A and HMIP-B, respectively).

## Discussion

*Aedes albopictus* feeds preferentially on human blood, and it is adapted to live in close proximity to humans, often in highly populated and urban areas [40, 41]. The species is an epidemiologically important vector for the transmission of many human pathogens, which makes it crucial to implement effective surveillance and control strategies [42]. Genetic epidemiological studies of human-mosquito networks are thus of great importance for a better understanding of the inherent risk associated with mosquito-borne arbovirus transmission. In spite of this, to our knowledge no one has ever proposed a valid genotyping method capable of simultaneously analyzing human and *Ae. albopictus* DNA for studies on human-mosquito interactions.

In the present study we developed for the first time an inexpensive, straightforward and universal STR-loci multiplex system capable of simultaneously amplifying human and *Ae. albopictus* STRs from blood-fed mosquitoes. The main novelty is that both vector and host DNA analysis (vector population genetics and human DNA fingerprinting) can be carried out at the same time starting from a single extraction of a single blood-fed mosquito, at lower cost than has previously been possible. Furthermore, one of the most remarkable methodological advantages of our assay comes from the ability to mix human and mosquito STRs: combining the microsatellites resulted in a reduction of the number of multiplexes used from four (one for human and three for *Ae. albopictus* STRs) to three (all of them combined human-mosquito multiplexes) and, consequently, allowed us to save laboratory work time and money during amplification and genotyping. Indeed, at present the total estimated cost per sample with our new system is around 15€, which covers all reagents and primers used for DNA extraction and STR amplification, as well as STR genotyping. In comparison, the cost per reaction of commercial kits for human STR genotyping employing the same 16 human STRs is at least approximately 25€ [26], bringing the total cost (DNA extraction plus genotyping expenses) to approximately 40€. Overall, our inexpensive STR method for analyzing human and mosquito DNA makes it possible to provide a fuller and more detailed picture of the transmission dynamics of mosquito-borne arboviruses. This makes the method particularly valuable when designing targeted policy interventions for controlling vector mosquito populations and reducing disease risk.

In the present study, 35 STR loci (16 human and 19 *Ae. albopictus* STRs) were tested for amplification and scoring reliability. All of them worked correctly in the laboratory environment. However, when we tested the protocol under natural non-optimal conditions, we detected amplification failures in the human microsatellite PentaD, which presented low amplification efficiency possibly due to the length of its products (376–449 bp). Amplicon length has been reported to directly affect amplification success of host DNA over time [36, 43], which is the reason why molecular analyses that target degraded DNA often use short amplicons [44], which are less sensitive to deterioration [36]. We also removed two other human STRs, vWA and D5S818, because of overlapping allele sizes. As a result, a total of 32 STRs were deemed useful, and their distribution among three mixed PCR multiplexes was successful, making it possible to retrieve mosquito and human genetic profiles simultaneously. Furthermore, while losing three human STRs, it is important to underscore that human profiles obtained by the remaining 13 loci were still unique and clearly differentiable.

In genetic analyses of field-collected specimens, it is important to keep in mind that the preservation method can have a direct effect on DNA integrity, and thus on the effectiveness of host DNA detection and fingerprinting [13]. In the present study, in which human DNA contained in *Ae. albopictus* blood meals is the target of the assay, it is crucial to protect remaining DNA template molecules from storage-associated degradation caused by oxidation, hydrolysis or radiation [45]. Lysis buffer and absolute ethanol were chosen as preservation agents here because of their many advantages, such as easy manipulation during field sampling, low risk of contamination, negligible cost and proven functionality and efficiency (e.g. [13, 36]). Although both methods returned amplification rates of 100% at 0 h of digestion, lysis buffer performed better than absolute ethanol in terms of allele PHs and clarity of electropherograms.

Similarly, post-feeding time delay has a negative effect on DNA integrity because of human DNA degradation caused by the mosquito’s digestive processes [20, 46]. Our results show a decreasing trend with time in allele detectability that can be represented as a descending sigmoidal curve. Although an important decrease in PHs was observed when the delay exceeded 24 h following the blood meal, complete human DNA profiles could be obtained, albeit with some degradation, up to 48 h. These observed patterns are consistent with the findings of numerous studies that report similar PH behavior [47] and the difficulty of retrieving complete or nearly complete host DNA profiles from around 36 h of digestion [17, 22, 48]. Furthermore, our results reinforce findings by Hiroshige et al. [47] that PH can be used as a proxy for post-feeding time. Nevertheless, it should be noted that mosquito samples used in the post-feeding time assay were stored in absolute ethanol, which could have had an effect on allele detectability. For future research, a reassessment of the evolution of DNA detectability over time using samples preserved in lysis buffer could lead to improved loci detectability.

In our field study, we could readily detect cases of multiple feedings (4/38 mosquitoes in which human DNA was found) and determine the number of different people bitten by a mosquito (either one or two; we did not detect any case of triple feeding) through the number of alleles detected at the different human STR loci. Furthermore, individual host profiles were obtained by looking at the relative PHs of human STRs, which is related to post-feeding time and to the amount of good quality DNA available for every human host [39, 47]. Nevertheless, it is important to keep in mind that difficulty in distinguishing different people in a single blood meal will depend on: (i) the level of DNA degradation of each person; (ii) the masking effect produced by higher alleles located in the same position; and (iii) the total number of people represented in the blood meal. For this reason, in multiple feeding cases we were able to obtain highly complete profiles only from the host with the highest PHs, whereas a decreasing tendency on the number of detected alleles was observed for the other hosts. This result suggests that multiple feedings can be a source of error for identification of the people bitten (i.e. host fingerprinting, especially with meals from > 2 people) but not for estimates of the biting frequency (i.e. number of different people bitten). Determination of mosquito biting frequency is crucial to achieve a correct understanding of mosquito-borne transmission dynamics [49]. Indeed, vector density alone (on which a good deal of research primarily focuses) cannot predict epidemic risk, as even low mosquito population levels might drive disease transmission through high multiple feeding rates. An integrative approach incorporating biting rates is thus needed for implementing efficient surveillance and control activities of disease-carrying mosquitoes.

## Conclusions

We provide a new, inexpensive and straightforward genotyping protocol that allows fast and reliable screening of both host and vector species simultaneously. It is intended to serve as a basis for future genetic epidemiological studies aimed at deeper insights into mosquito-human interactions and vector-borne disease ecology, with the ultimate goal of improving evaluation of epidemiologic risk as well as security and surveillance measures against mosquito-borne pathogens. The genotyping system we propose can also be further adapted to other vector species living in close contact with humans.

Additionally, our study suggests that lysis buffer performs better than absolute ethanol for preserving *Ae. albopictus* blood meals. The results also show that it is possible to obtain complete human DNA profiles up to 48 h post-feeding in mosquito blood meals. Finally, our multiple feeding analysis confirms that STR peak heights are tightly linked to post-feeding time and can be used to derive the number of different people bitten by a mosquito.

### Supplementary Information

**Additional file 1: Figure S1.** Map of the study area. Main map shows the Iberian Peninsula, with the Blanes study site marked just above Barcelona. Made with Natural Earth, free vector and raster map data from Natural Earth (https://www.naturalearthdata.com). Inset map shows orthophoto of Blanes, with the Marimurtra Botanical Garden field site marked in yellow and the CEAB laboratory marked in blue. Map derived from the Orthophoto of Catalonia 1:5.000 of the Institut Cartogràfic i Geològic de Catalunya (ICGC), used under a CC BY 4.0 license.

## Data Availability

The datasets supporting the conclusions of this article are available in the Zenodo repository: https://doi.org/10.5281/zenodo.8328859.

## Abbreviations

ANOVA: Analysis of variance
PBS: Phosphate buffered saline
PCoA: Principal Coordinates Analysis
PHs: Peak heights
STRs: Short tandem repeats
